# Bone-related Circulating MicroRNAs miR-29b-3p, miR-550a-3p, and miR-324-3p and their Association to Bone Microstructure and Histomorphometry

**DOI:** 10.1038/s41598-018-22844-2

**Published:** 2018-03-20

**Authors:** Xaver Feichtinger, Christian Muschitz, Patrick Heimel, Andreas Baierl, Astrid Fahrleitner-Pammer, Heinz Redl, Heinrich Resch, Elisabeth Geiger, Susanna Skalicky, Rainer Dormann, Fabian Plachel, Peter Pietschmann, Johannes Grillari, Matthias Hackl, Roland Kocijan

**Affiliations:** 1 Ludwig Boltzmann Institute for Experimental and Clinical Traumatology, Vienna, Austria; 2 St. Vincent Hospital – Medical Department II, The VINFORCE Study Group, Academic Teaching Hospital of the Medical University of Vienna, Vienna, Austria; 3 AUVA Trauma Center Meidling, Vienna, Austria; 4 Austrian Cluster for Tissue Regeneration, Vienna, Austria Department of Traumatology, Medical University of Vienna, Vienna, Austria; 5 Karl Donath Laboratory for Hard Tissue and Biomaterial Research, Department of Oral Surgery, Medical University of Vienna, Vienna, Austria; 6 Department of Statistics and Operations Research, University of Vienna, Vienna, Austria; 7 Department of Internal Medicine, Division of Endocrinology and Diabetology, Medical University of Graz, Graz, Austria; 8 Medical Faculty of Bone Diseases, Sigmund Freud University, Vienna, Austria; 9 TAmiRNA GmbH, Vienna, Austria; 10 Department of Pathophysiology and Allergy Research, Center for Pathophysiology, Infectiology and Immunology, Medical University of Vienna, Vienna, Austria; 11 Christian Doppler Laboratory on Biotechnology of Skin Aging, Department of Biotechnology, BOKU - University of Natural Resources and Life Sciences Vienna, Vienna, Austria

## Abstract

The assessment of bone quality and the prediction of fracture risk in idiopathic osteoporosis (IOP) are complex prospects as bone mineral density (BMD) and bone turnover markers (BTM) do not indicate fracture-risk. MicroRNAs (miRNAs) are promising new biomarkers for bone diseases, but the current understanding of the biological information contained in the variability of miRNAs is limited. Here, we investigated the association between serum-levels of 19 miRNA biomarkers of idiopathic osteoporosis to bone microstructure and bone histomorphometry based upon bone biopsies and µCT (9.3 μm) scans from 36 patients. Four miRNAs were found to be correlated to bone microarchitecture and seven miRNAs to dynamic histomorphometry (p < 0.05). Three miRNAs, namely, miR-29b-3p, miR-324-3p, and miR-550a-3p showed significant correlations to histomorphometric parameters of bone formation as well as microstructure parameters. miR-29b-3p and miR-324-p were found to be reduced in patients undergoing anti-resorptive therapy. This is the first study to report that serum levels of bone-related miRNAs might be surrogates of dynamic histomorphometry and potentially reveal changes in bone microstructure. Although these findings enhance the potential value of circulating miRNAs as bone biomarkers, further experimental studies are required to qualify the clinical utility of miRNAs to reflect dynamic changes in bone formation and microstructure.

## Introduction

Idiopathic osteoporosis (IOP) is characterized by low-trauma fracture occurrence in young men and premenopausal women without any identifiable cause for systemic bone loss or secondary osteoporosis^1^. An osteoblast dysfunction has been suggested for both genders^2,3^. Moreover, an uncoupling of bone formation and resorption has been suggested in women with IOP^2,3^.

The assessment of both bone quality and the prediction of fracture risk is complex in IOP as areal bone mineral density (aBMD) and established bone turnover markers (BTM) do not sufficiently explain the increased fracture risk^4,5^.

Bone microstructure is one of the main contributors of bone strength. The association between impaired trabecular and cortical bone architecture to low-trauma fractures was previously reported. High-resolution peripheral quantitative computed tomography (HR-pQCT) studies suggest relationships between fracture risk and bone microstructure, largely independent of BMD. It has been demonstrated that vertebral and peripheral fractures were associated with deteriorations in trabecular and cortical bone including trabecular bone volume, cortical thickness, trabecular number, and trabecular thickness^6–8^.

High cortical porosity was further proposed to be a viable explanation for low-trauma fracture occurrence and has been the subject of earnest investigation in recent years^9^. Cortical porosity seems to be related to peripheral fracture risk in both postmenopausal and premenopausal women.

A novel approach for diagnosis and follow-up of IOP are circulating microRNAs (miRNAs) which are present in human serum. miRNAs are small non-coding RNAs acting as post-transcriptional regulators of gene expression. Several miRNAs were reported to regulate bone formation, -resorption, remodeling, and differentiation of bone cells^10^. Specific signatures of circulating miRNAs were identified after acute osteoporotic fractures: in postmenopausal patients with prevalent fracture, as well as in premenopausal and male IOP and postmenopausal women with low-trauma fractures^11–13^. Despite group differences, the latter study reported a set of 19 miRNAs, which was consistently regulated in all three groups (three up-regulated and 16 down-regulated miRNAs in the fracture groups). These miRNAs were excellent discriminators of patients with low-traumatic fractures, regardless of age and gender.

We hypothesized that the complex pattern of IOP could be characterized by (i) bone microstructure and that there would be (ii) a significant association to serum levels of previously reported miRNAs.

Accordingly, the primary objective of this study was to evaluate associations between 19 recently identified bone-related circulating miRNAs, bone microstructure, and bone histomorphometry in patients with male and premenopausal IOP as well as postmenopausal osteoporosis with low-traumatic fractures^11^.

Secondary objectives were to (i) compare trabecular and cortical bone microstructure between male, premenopausal as well as postmenopausal osteoporosis, and to (ii) correlate between histomorphometry and bone microstructure by µCT at a high-resolution level.

## Materials and Methods

### Study Design

In this cross-sectional study bone microstructure, histomorphometry, and miRNA expression were assessed in male and female patients with idiopathic osteoporosis as well as postmenopausal osteoporosis with low traumatic fractures. All patients were recruited at the St. Vincent Hospital Vienna (Medical Department II for Rheumatology and Bone Diseases), a specialist referral center for bone diseases. Microstructure analysis was performed by the Ludwig Boltzmann Institute for Experimental and Clinical Traumatology in Vienna; bone histomorphometry by the Medical University of Graz (Department of Internal Medicine, Division of Endocrinology and Diabetes); and miRNA analysis by the University of Natural Resources and Life Sciences Vienna (Department of Biotechnology) and TAmiRNA GmbH. The study was approved by the ethics committee of the St. Vincent Hospital Vienna (approval number: 201501_EK04) and was conducted in accordance with the Declaration of Helsinki. All participants signed written informed consent documents.

### Subjects

Men and women with idiopathic osteoporosis or postmenopausal osteoporosis and at least one peripheral or vertebral low-trauma fracture were included in the present study. All subjects were Caucasian and participated in the primary study on circulating miRNA signatures and low-traumatic fractures^11^. Low-traumatic fractures were considered as fractures that occurred without any or after minor trauma^14^. Vertebral fractures were assessed by lateral and antero-posterior digital X-rays of the lumbar and thoracic spine. Peripheral fractures were evaluated by questionnaire. The time span between the last fracture to study-related activities had to be at least six months in order to prevent the potential influence of fracture healing on miRNA profiles.

Patients were classified as having idiopathic osteoporosis if they were (i) men or premenopausal women aged < 50 years who (ii) had sustained low-traumatic fractures but had an otherwise normal medical history, (iii) or had osteopenic or normal BMD values but had sustained low-traumatic fractures. Postmenopausal women were included in the study if they had sustained (i) low-traumatic fractures regardless of their T-score and (ii) had no obvious reason for secondary osteoporosis mentioned in the exclusion criteria^11^. All patients who fulfilled the criteria were included consecutively.

Exclusion criteria included: (i) secondary causes for osteoporosis including diabetes mellitus type 1 and 2, inflammatory diseases (including rheumatoid arthritis, psoriatic arthritis, systemic lupus erythematosus, Crohn’s disease, ulcerative colitis), COPD, chronic kidney and liver dysfunction, systemic glucocorticoid use and glucocorticoid induced osteoporosis, eating disorders, HIV-infections and genetic disorders affecting bone such as osteogenesis imperfecta, Ehlers-Danlos-syndrome, hyophosphatasia, hypophospahtemic rickets and fibrous dysplasia; (ii) malignant diseases including plasmocytosis and lymphoma; (iii) previous or current teriparatide or strontium ranelate therapy.

Exclusion criteria were determined by clinical investigations, bone biopsy and blood analysis (including full blood count, parameters of liver and renal function, serum electrophoresis, BTMs, vitamin D, electrolytes). aBMD at the lumbar spine and hip was assessed by DXA. The results have been reported elsewhere^11^. The patient’s medical history, previous fractures including trauma history as well as current and previous medicines including bone-related therapy were recorded. For further analysis patients were divided into three groups (i) premenopausal IOP, (ii) male IOP and (iii) postmenopausal osteoporosis. The fracture risk assessment score (FRAX®) was calculated for all patients with the assumption that all patients were 40 years of age or older. For younger patients, age was considered as “40 years” to allow for calculation.

### Bone Biopsies

Transiliac crest biopsies were performed in all patients by two experienced clinicians (CM, RK). Prior to procedure (24 and 8 days beforehand), patients received 250 mg tetracycline hydrochloride orally every 6 h for 3 consecutive days. Biopsies were performed by manual drill using a Bordier-type trephine (7.5 mm). Each patient underwent two parallel-oriented transiliac biopsies. The obtained samples were stored and transported in 70% ethanol or RNA-later® for subsequent analysis.

### Bone Histomorphometry Analysis

For histomorphometric analysis all biopsies were fixed in ethanol, dehydrated and embedded in polymethyl methacrylate (PMMA). Histological sections (2–5 µm) were cut from the blocks and measured under a light microscope either unstained (under fluorescent light) or stained (Goldner or toluidineblue) using a bone histomorphometry system (OsteoMeasure System; OsteoMetricsTM, Decatur, GA 30030, USA)^3,15^. In total, three stained and 5 unstained sections were analyzed and the results were averaged. The entire periosteal and endocortical bone envelopes were measured under 100 × magnification. Evaluation of the intracortical resorbing osteon were carried out under 200 × magnification. Dynamic and static assessments were performed by an impartial clinician^15^.

All measured and derived variables were expressed in accordance with the histomorphometric criteria recommended by the subcommittee on bone histomorphometry of the American Society for Bone and Mineral Research^16^. The following parameters were assessed: trabecular bone volume (BV/TV), bone surface (BS/BV), osteoid surface (OS/BS), eroded surface (ES/BS), quiescent surface (QS/BS), trabecular number (Tb.N), trabecular thickness (Tb.Th), trabecular separation (Tb.Sp), mineralizing surface (MS/BS), mineral apposition rate (MAR – calculated from the interlabel distance and number of days between labeling), and bone formation rate (BFR/BS). BFR was calculated using all visible double-labeled and one half of the single-labeled surfaces given as cubic micrometers per square micrometers per day. Labels were present in all samples analyzed.

### Bone Microstructure Assessment

Trabecular and cortical bone microstructure was evaluated by µCT imaging system (µCT 50, SCANCO Medical AG, Brüttisellen, Switzerland). The µCT combines submicron pixel size with a large field-of-view and is equipped with a 5–30 µm (4–18 W) spot X-ray tube as a source. The tube was operated at 70 kVp. Due to the bone sample size, the scans were performed at an isotropic nominal resolution of 9.3 µm. The long axis of the biopsy specimen was oriented along the rotation axis of the scanner. All samples were scanned concurrently.

Trabecular and cortical microarchitecture analyses were performed using ImageJ with the BoneJ plugin^17^. A semi-automated approach was used to define cortical and trabecular area (see Supporting Fig. 1). Cortical microstructure analysis was performed at the outer cortex. The volume of interest (VOI) was manually drawn and interpolated using the ROI interpolation function in ImageJ. The VOI was adapted for all slices. A mean of 900 and 650 slices were analyzed per bone biopsy for trabecular and cortical bone, respectively. The images scanned using the SCANCO µCT were calibrated by means of the SCANCO calibration phantom. The images were adjusted with 375 mg HA/cm³.

**Figure 1:**
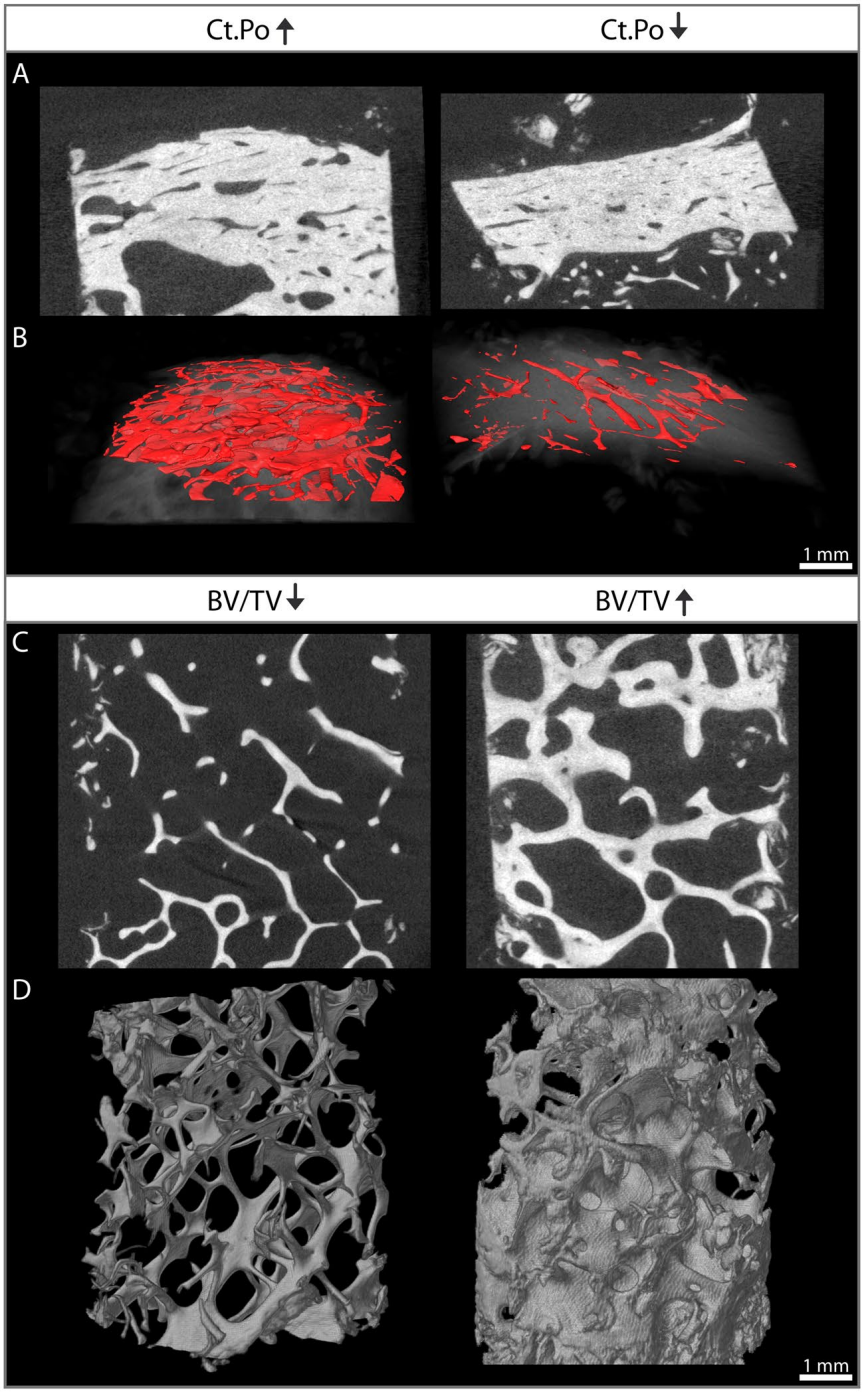
Cortical and trabecular bone microstructure in male idiopathic osteoporosis. Images of two male patients were chosen for this figure to highlight the heterogeneity of bone quality within the study groups. The variation of bone structure parameters was similar in premenopausal and postmenopausal women. The heterogeneity of bone microstructure within the subgroups is further reflected by the high standard deviation and the absence of significant findings between the subgroups (Table 2). Left, high cortical porosity (Ct.Po) and low trabecular bone volume (BV/TV); right, low Ct.Po and high BV/TV. (**A**) Ct.Po, 2-D analysis; (**B**) Ct.Po. 3-D reconstruction; (**C**) BV/TV, 2-D analysis; (**D**) BV/TV, 3-D reconstruction.

Trabecular microstructure parameters, including trabecular bone volume fraction (BV/TV), mean trabecular thickness (Tb.Th mean, µm), and maximum trabecular thickness (Tb.Th max, µm) were established. Cortical parameters including cortical porosity (Ct.Po, %), mean cortical pore diameter (Pore DM mean) and maximum cortical pore diameter (Pore DM max) were analyzed.

### microRNA analysis

The analysis of circulating miRNAs in serum samples was performed by TAmiRNA (Vienna, Austria) using previously described protocols^11–13^. Quantitative data for 19 bone-related miRNAs, recently found to be optimal discriminators of low-traumatic fractures in both IOP and postmenopausal osteoporosis, were used in the current study for correlation to bone histomorphometry and bone microstructure^11^. In brief, serum microRNA analysis is based on a reverse-transcription quantitative PCR assay (RT-qPCR) on total RNA isolated from 200 µl of serum. Total RNA was extracted using the miRNeasy RNA extraction kit (Qiagen, Germany). Three spike-in controls were added to the lysis buffer, which enables the monitoring of microRNA recovery during extraction. Total RNA was re-suspended in 30 µl of nuclease-free water. Conversion into cDNA was performed using the osteomiR™ kit, which enables universal transcription of all miRNA species. Altogether, 2 µl of total RNA were converted into cDNA at 42 °C for 60 minutes. Non-human cel-miR-39 was added to this reaction to monitor the efficiency and to detect the presence of enzyme inhibitors. Targeted qPCR amplification was performed in primer-coated 96-well plates, which are part of the osteomiR™ kit, and ExilENT SYBR® Green Master Mix. The reaction volume was 10 µl. The reaction was started by an activation step at 95 °C for 10 minutes. This was followed by 45 PCR cycles including two steps: denaturation (95 °C, 10 s), along with combined annealing and extension (60 °C, 60 s). A PCR positive control is included on each plate to monitor variance in PCR efficiency.

Raw data from PCR amplification were converted into Cq-values using the second-derivative method as implemented in the osteomiR™ software. Samples of insufficient quality or **those** affected by hemolysis were removed from the analysis^11^. Raw Cq-values were subsequently normalized using an in-house script developed to remove technical variance using spike-in controls assays and imputation of missing values.

### Statistical analysis

All patients who met the inclusion criteria and (i) had no exclusion criteria and (ii) underwent bone biopsy were included consecutively. Thus, a sample size calculation was not performed. Quantitative clinical characteristics, bone microarchitecture, and bone histomorphometry were compared between groups (premenopausal, postmenopausal, males) by overall F-Tests and t-tests for pairwise group comparisons. For binary and nominal measures, Fisher’s exact test and its extension for >2 × 2 tables were applied (Tables 1 and 2).

**Table 1:** Clinical characteristics of the study cohort.

	Pre-MP	Post-MP	MIO	p-values			
				Pre-MP vs. Post-MP	Pre-MP vs. MIO	Post-MP vs. MIO	overall
No. of patients	10	10	16				
Age (yr)	39.0 ± 8.6	59.0 ± 11.4	43.7 ± 11.1	<*0.001*	0.280	*0.001*	<*0.001*
Height (cm)	165.8 ± 4.0	164.6 ± 4.6	176.6 ± 7.2	0.645	<*0.001*	<*0.001*	<*0.001*
Weight (kg)	65.3 ± 20.0	69.0 ± 12.9	76.6 ± 15.6	0.616	0.097	0.260	0.217
Body Mass Index (kg/m2)	23.8 ± 8.0	25.5 ± 4.7	24.5 ± 4.3	0.492	0.761	0.645	0.783
Vertebral fractures, mean	1.9 ± 2.6	1.8 ± 2.0	1.9 ± 2.1	0.920	0.978	0.934	0.994
Peripheral fractures, mean	1.3 ± 2.4	2.0 ± 3.2	3.3 ± 5.9	0.730	0.290	0.495	0.542
FRAX®, major osteoporotic	11.0 ± 3.6	16.4 ± 6.9	14.3 ± 13.7	0.242	0.415	0.624	0.490
FRAX®, hip fracture	3.6 ± 3.2	5.0 ± 6.1	8.1 ± 14.5	0.765	0.286	0.458	0.527
Smoking (%)	30	0	67	0.211	0.111	<*0.001*	*0.001*
Alcohol (%)	0	10	7	1.000	1.000	1.000	1.000
Previous treatment (%)	50	73	31	0.650	0.069	*0.016*	*0.026*
Lactose intolerance (%)	10	0	25	1.000	0.617	0.136	0.243
Family history (%)	30	30	13	1.000	0.358	0.358	0.535

Pre-MP, premenopausal idiopathic osteoporosis; Post-MP, postmenopausal osteoporosis; MIO, male idiopathic osteoporosis; FRAX, fracture risk assessment tool reflecting the ten year probability of fracture.

**Table 2:** Bone histomorphometry and microstructure by µCT in the study cohort.

	Pre-MP	Post-MP	MIO	p-values			
				Pre-MP vs. Post-MP	Pre-MP vs. MIO	Post-MP vs. MIO	overall
**Bone Histomorphometry**							
BV/TV (%)	17.5 ± 4.4	17.0 ± 8.9	16.7 ± 9.0	0.889	0.804	0.938	0.969
BS/BV (1/mm)	16.7 ± 2.8	18.6 ± 5.3	19.6 ± 7.3	0.527	0.255	0.709	0.516
OS/BS (%)	10.9 ± 8.1	6.2 ± 5.2	10.3 ± 10.0	0.289	0.867	0.317	0.512
ES/BS (%)	8.02 ± 7.75	4.15 ± 4.47	4.38 ± 4.29	0.180	0.138	0.931	0.262
QS/BS (%)	81.1 ± 12.7	89.6 ± 7.2	84.4 ± 12.3	0.150	0.511	0.337	0.346
Tb. N (1/mm)	1.44 ± 0.33	1.39 ± 0.52	1.39 ± 0.38	0.821	0.787	0.997	0.958
Tb. Th (µm)	122.3 ± 19.0	114.7 ± 30.3	117.0 ± 46.6	0.685	0.736	0.896	0.909
Tb. Sp (µm)	605.1 ± 161.0	728.7 ± 432.7	648.3 ± 216.3	0.368	0.709	0.523	0.658
MS/BS (%)	4.78 ± 4.76	2.27 ± 2.32	2.00 ± 2.20	0.168	0.080	0.873	0.180
MAR (µm/day)	0.85 ± 0.68	0.80 ± 1.32	1.74 ± 2.81	0.967	0.385	0.383	0.570
BFR/BS (µm^3^/µm^2^/day)	25.4 ± 35.2	13.1 ± 25.0	15.8 ± 22.1	0.452	0.477	0.856	0.695
**Bone Microstructure**							
Cortical Bone							
Ct. Po (%)	7.2 ± 6.7	6.0 ± 2.4	6.9 ± 4.0	0.553	0.868	0.628	0.821
Ct. Pore DM Mean (µm)	88.1 ± 34.2	80.4 ± 23.5	83.9 ± 31.0	0.571	0.731	0.781	0.849
Ct. Pore DM Max (µm)	237.5 ± 75.8	207.8 ± 57.2	236.8 ± 117.2	0.477	0.985	0.448	0.701
Trabecular Bone							
BV/TV (%)	14.0 ± 3.1	16.3 ± 7.7	16.6 ± 9.2	0.491	0.397	0.932	0.671
Tb. Th (µm)	151.1 ± 17.7	168.8 ± 39.3	159.2 ± 50.7	0.341	0.630	0.562	0.630
Tb. Th Max (µm)	387.6 ± 61.6	373.5 ± 97.2	375.3 ± 116.2	0.750	0.759	0.964	0.938

BV/TV, trabecular bone volume; BS/BV, bone surface; OS/BS, osteoid surface, ES/BS, eroded surface; QS/BS, quiescent surface; Tb.N, trabecular number; Tb.Th, trabecular thickness; Tb.Sp, trabecular separation; MS/BS, mineralizing surface; MAR, mineral apposition rate, BFR/BS, bone formation rate; Ct.Po, cortical porosity, Ct.Pore DM, cortical pore diameter.

The relationships between serum levels of 19 circulating miRNAs, bone microarchitecture, and bone histomorphometry were estimated using Spearman correlation coefficients. P-values together with 95% confidence intervals for correlation coefficients were reported (Table 3, Supporting Table 1, Supporting Data 1).

**Table 3:** Correlations between Histomorphometry and Microstructure by µCT.

		Histomorphometry			
		BV/TV (%)	Tb. N (1/mm)	Tb. Th (µm)	Tb. Sp (µm)
µ**CT**	**Ct. Po (%)**	0.27 [−0.14, 0.62]	−0.12 [−0.51, 0.32]	**0.48** [**0.08, 0.77]**	0.02 [−0.41, 0.41]
	**Ct. Pore DM Mean (µm)**	0.19 [−0.22, 0.57]	−0.1 [−0.5, 0.32]	0.34 [−0.05, 0.67]	0.02 [−0.4, 0.43]
	**Ct. Pore DM Max (µm)**	0.38 [0, 0.69]	0.08 [−0.35, 0.5]	**0.47** [**0.08, 0.76]**	−0.16 [−0.55, 0.26]
	**BV/TV (%)**	***0.77*** [***0.54, 0.91***]	**0.5** [**0.15, 0.78**]	***0.69*** [***0.4, 0.86***]	**−0.6** [**−0.83, −0.26**]
	**Tb. Th (µm)**	***0.57*** [***0.29, 0.75***]	0.14 [−0.25, 0.49]	***0.71*** [***0.43, 0.88***]	−0.25 [−0.55, 0.12]
	**Tb. Th Max (µm)**	***0.63*** [***0.32, 0.83***]	0.3 [−0.08, 0.6]	***0.66*** [***0.35, 0.86***]	**−0.4** [**−0.67, −0.04**]

**bold**, significance < 0.05; ***Italic and bold***, significance < 0.005; BV/TV, trabecular bone volume; Tb.N, trabecular number; Tb.Th, trabecular thickness; Tb.Sp, trabecular separation; Ct.Po, cortical porosity, Ct.Pore DM, cortical pore diameter. Spearman correlation + 95%-CI.

As to the question of whether adjusting for anti-resorptive pre-treatment or group (premenopausal, postmenopausal, males) influences the relationship between circulating miRNAs and bone parameters, we constructed step-wise multiple linear regression models. F-values, p-values and multiple testing adjusted p-values in the form of false-discovery rates. (Supporting Fig. 2).

**Figure 2:**
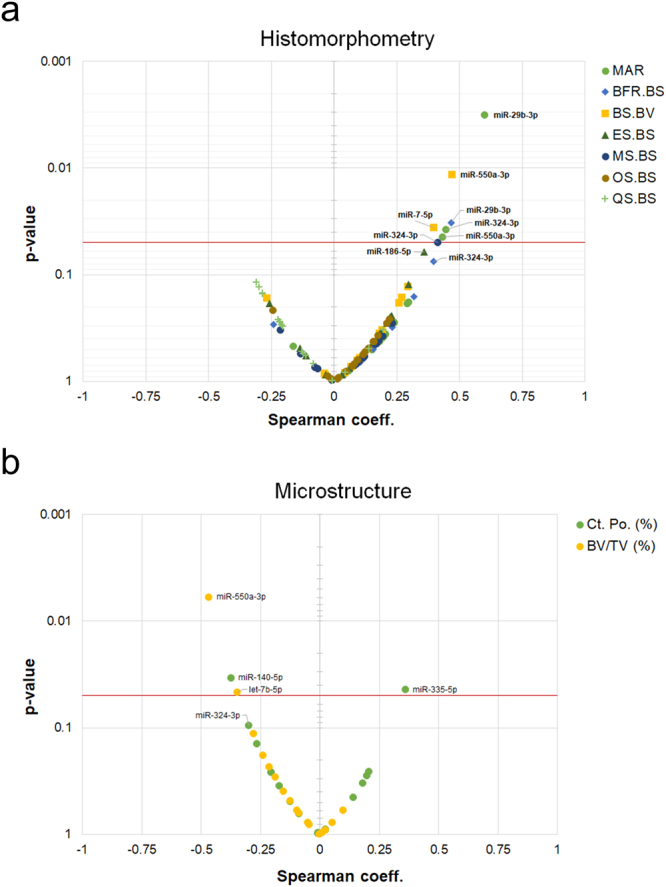
Volcano plot of Spearman coefficients versus p-values. (**a**) Bone histomorphometry: seven bone histomorphometric parameters were correlated to serum levels of 19 microRNAs. Spearman coefficients (x-axis) and p-values (y-axis) are shown. Labels were added for those microRNAs where p-values for association were equal or below 0.05. (**b**) bone microstructure: an analogous analysis was performed for trabecular (BV/TV) and cortical porosity (Ct. Pos.). Likewise, microRNAs with p-values < 0.05 are labelled in the plot.

All tests were two-sided and p-values less than 0.05 were considered statistically significant. Statistical analyses were performed with the statistical software R version 3.3.1^18^.

## Results

### Clinical characteristics

Thirty-six adult patients with low-traumatic fractures (mean age 46.6 ± 13.0 years) were included in the study: 10 premenopausal women (mean age 39.0. ± 8.6 years), 10 postmenopausal women (mean age 59.0 ± 11.4 years) and 16 men (mean age 43.7 ± 11.1 years). While age and height were significantly different, body mass index (BMI) was comparable between the groups (Table 1). Vertebral fractures and non-vertebral low-traumatic fractures were observed in 8 patients (22%). 18 patients (50%) suffered exclusively vertebral fractures. 10 patients (28%) suffered exclusively non-vertebral fractures. The mean number of fractures was 4.2. The distribution of fractures and the calculated FRAX® score were similar between groups. Pre-treatment with anti-resorptive drugs (bisphosphonates, denosumab, n = 13) was observed in 32% of patients. The overview of the clinical characteristics of the study population is shown in Table 1. Neither BTMs, nor aBMD, nor clinical risk factors explained fracture occurrence in our patients as had been reported previously^11^.

### Bone Histomorphometry and Microstructure between groups

No differences were found regarding static and dynamic histomorphometry parameters between premenopausal IOP, postmenopausal osteoporosis, and male IOP. In addition, comparable cortical and trabecular bone microstructure parameters by µCT were found between the three groups (see Table 2). In contrast, microstructure parameters were distributed heterogeneously within the groups (see Fig. 1).

Patients with exclusively vertebral fractures had significantly lower BV/TV (p = 0.01) and Tb.N. (p = 0.01) as well as higher Tb.Sp. (p = 0.03) compared to patients with peripheral fractures exclusively. Peripheral fractures were associated to significantly lower ES/BS (p = 0.01). Cortical porosity and Ct. Pore DM Max (µm) were increased in patients with peripheral fractures when compared to vertebral fractures (both p = 0.14). In conclusion, fractures of vertebral bodies - which mainly consist of trabecular bone - were related to impaired trabecular bone microstructure. In contrast, fractures of peripheral bones - which mainly consist of cortical bone - were associated to deteriorations of cortical parameters. Overview of histomorphometry and microstructure parameters is presented in Table 2.

### Correlations of Microstructure between Histomorphometry and µCT

Highly significant, positive correlations were found between the trabecular parameters by histomorphometry and µCT: BV/TV (histo) and BV/TV (µCT), BV/TV (histo) and Tb.Th (µCT), Tb.N (histo) and BV/TV (µCT), Tb.Th (histo) and BV/TV as well as Tb.Th (µCT). Tb.Sp (histo) was negatively correlated to BV/TV and Tb.Th (µCT). In summary, as a proof of concept, highly significant correlations were found between the trabecular parameters measured by histomorphometry and μCT. Moreover, significant associations between Tb.Th (histo) and cortical porosity and the maximum cortical pore diameter (µCT) were evaluated (see Table 3).

### Correlations between miRNAs, Bone Histomorphometry and Microstructure

To date, little is known about whether levels of circulating miRNAs might be associated to the structural and dynamic parameters of bone. To explore these possible correlations, we first investigated whether anti-resorptive treatment (ART) or subgroup (i.e. male, premenopausal, or postmenopausal) could influence the association between miRNAs and dynamic histomorphometry and microstructural parameters. Step-wise multiple linear regression models were constructed. F-values, p-values and multiple testing adjusted p-values in the form of false-discovery rates were obtained for two regression models assuming either offsets between the regression lines obtained for each subgroup (Model 1) or differences in the nature of regression (i.e. slope) between the subgroups (Model 2). This analysis identified only weak significant effects of subgroup or ART on the association of miRNAs and bone parameters, which disappeared completely after adjustments for multiple testing (false-discovery rate, FDR) had been made (Supporting Fig. 2). Therefore, correlation analysis was performed on the complete dataset of 36 patients with available microstructure and bone histomorphometry data.

For visual inspection of the magnitude and significance of associations, volcano plots were graphed showing the relationship between the Spearman rank-order correlation coefficient and significance level (Fig. 2).

**Figure 3:**
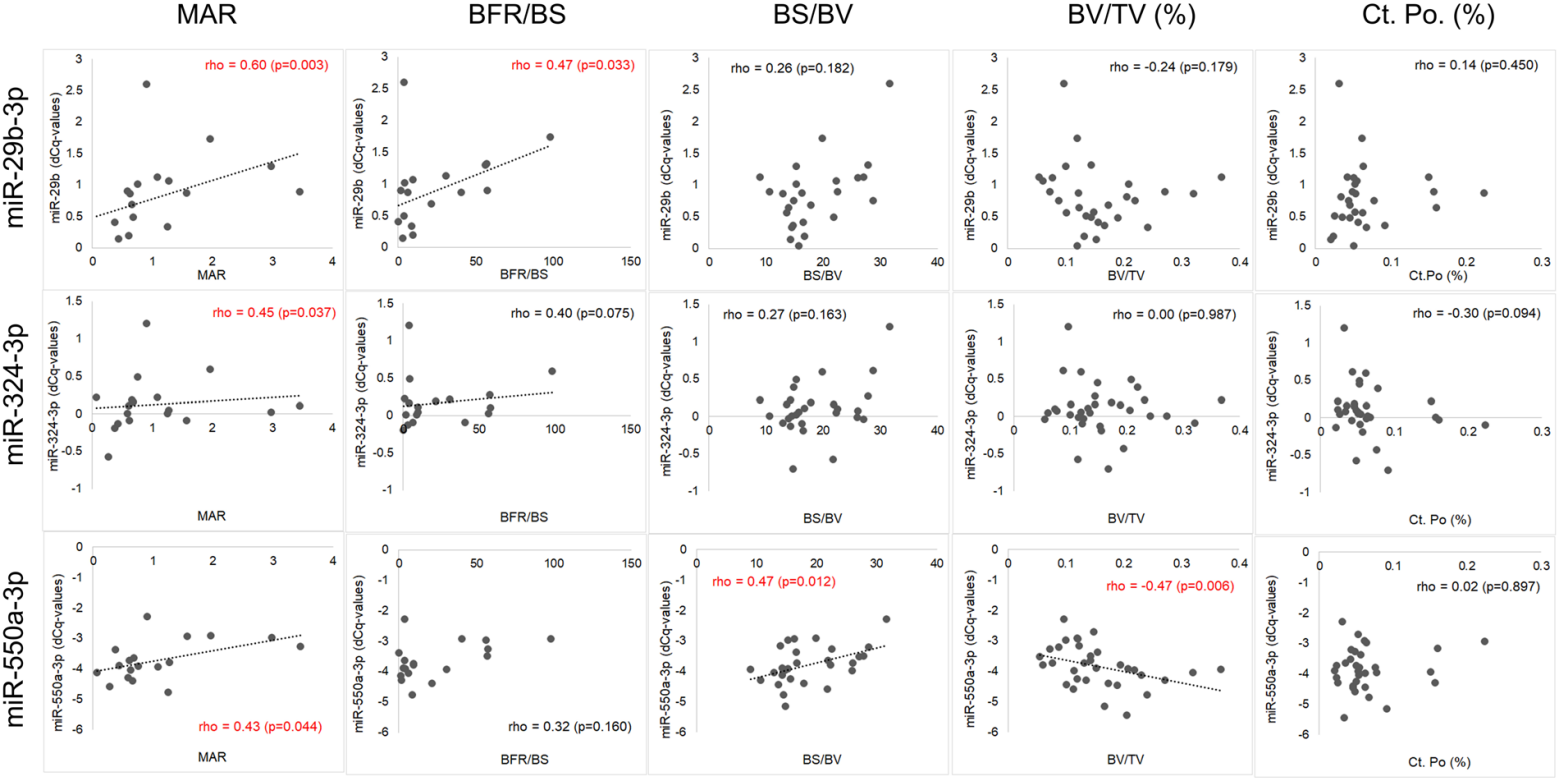
Scatterplots. Values and relationships between three miRNAs, miR-29b-3p, miR-324-3p, and miR-550a-3p, on the y-axis, and selected parameters from bone histomorphometry (MAR, BFR/BS, BS/BV) and microstructure (BV/TV, Ct. Po) on the x-axis, are shown. Spearman correlation coefficients and significance levels are shown in each plot. Red color signals p < 0.05. Linear trend lines have been added to plots with p < 0.05.

In total, seven significant pairwise correlations between miRNAs and histomorphometry parameters were observed indicating predominately positive associations (Fig. 2A). Four significant associations between miRNAs and microstructure were found (Fig. 2B). Three miRNAs, miR-29b-3p, miR-324-3p, and miR-550a-3p showed significant correlations to multiple parameters such as MAR, BFR/BS, BS/BV as well as BV/TV (Fig. 3). All three miRNAs were positively correlated to MAR (p < 0.05). In addition, miR-29b-3p and miR-324-3p were positively correlated to BFR/BS, and miR-550a-3p to BS/BV, respectively. In terms of microstructure, we observed a significant negative association between changes in trabecular BV/TV and miR-550a-3p (r = −0.47, p = 0.006), and with miR-29b-3p by trend (r = −0.24, p = 0.18). Changes in cortical porosity were found to be negatively correlated to changes in miR-324-3p, although not significantly (r = −0.30, p = 0.094). In conclusion, miRNAs miR-29b-3p, miR-324-3p, and miR-550a-3p were significantly associated to dynamic histomorphometric parameters, reflecting bone formation. In contrast, no associations were found between those miRNAs and parameters of bone resorption, such as ES/BS.

**Figure 4:**
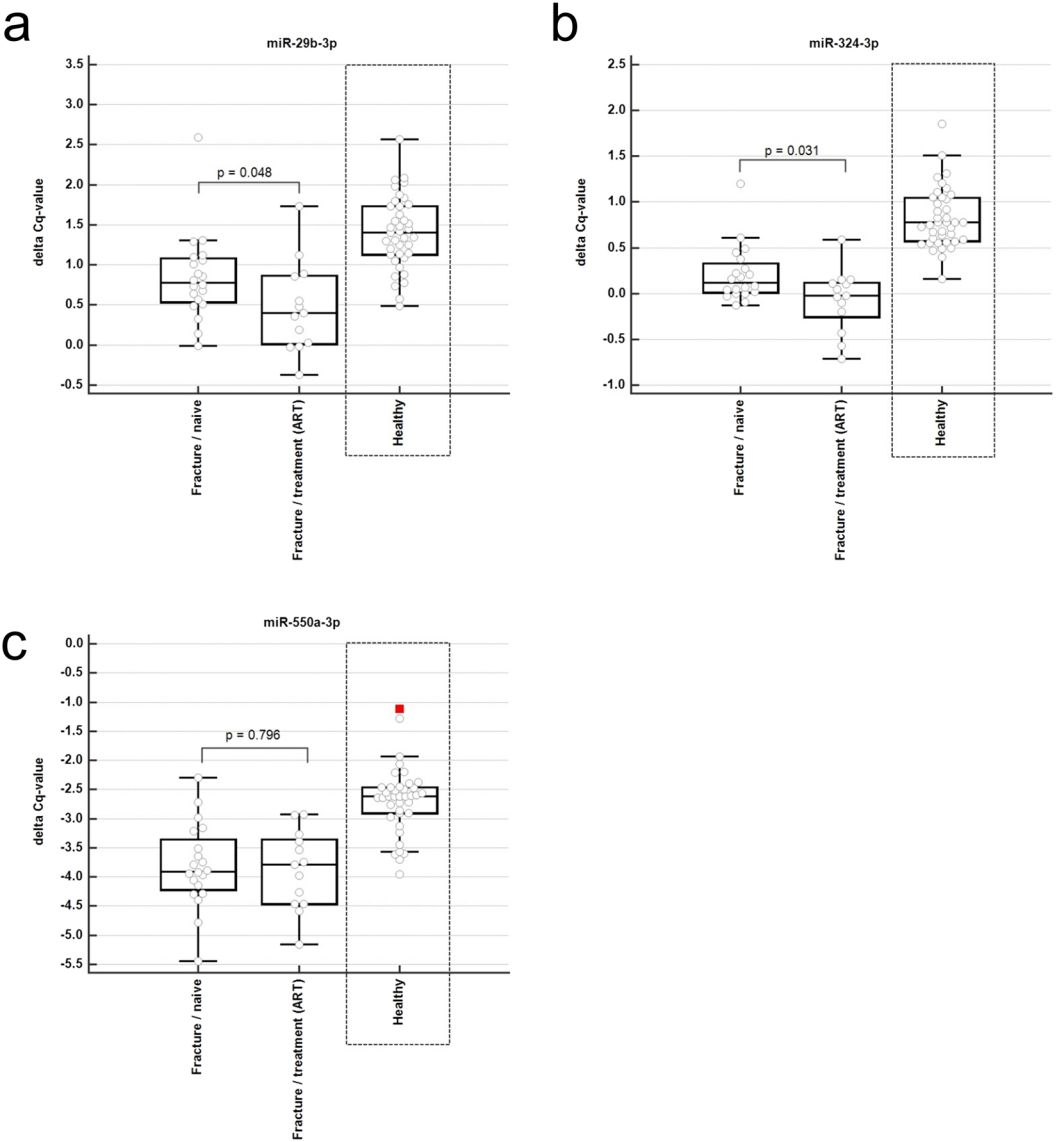
Boxplots. Distribution of the normalized delta-Cq values of (**a**) miR-29b-3p, (**b**) miR-324-3p, and (**c**) miR-550a-3p between treatment-naïve (Fracture/naive, n = 20) and bisphosphonate pre-treated subjects (Fracture/treatment (ART), n = 13) and are shown. As a reference, the normalized delta Cq-values in a matched healthy reference group are shown, which has been previously described (ref.^11^). P-values for the difference between treated and untreated subjects with fracture derived from non-parametric Kruskal-Wallis tests.

Although these associations were independent of subgroup and ART, we tested whether serum levels of miR-29b-3p, miR-324-3p and miR-550a-3p were different in patients post treatment, compared to untreated patients using Kruskal-Wallis test for non-parametric data (Fig. 4). We observed significant down-regulation for miR-29b-3p (p = 0.048) and miR-324-3p (p = 0.031) in patients undergoing ART compared to treatment-naïve patients. Serum levels of miR-550a-3p were not significantly altered under ART.

## Discussion

This study is the first to report several significant associations between circulating miRNAs and dynamic histomorphometry parameters in premenopausal women and men with IOP as well as postmenopausal women with low-traumatic fractures. In comparison, the associations between circulating miRNAs and static bone microstructure were found to be weaker, suggesting that dynamic changes in bone turnover are better reflected in the levels of circulating miRNAs.

miR-29b-3p is a well-characterized and important regulator of osteoblast formation by directly fine-tuning the expression of several structural bone matrix genes such as collagens (COL1A1, COL5A3, COL4A2) and the matricellular gene SPARC (osteonectin) during osteoblast formation^19^. Overexpression of miR-29b-3p was repeatedly shown to accelerate bone formation through direct down-regulation of factors inhibiting osteogenic differentiation including β-Catenin binding protein (CTNNBIP1), a negative regulator of WNT-signaling^19,20^. At this point we can report a significant positive correlation between circulating levels of miR-29b-3p and two important dynamic measures of bone formation (MAR, BFR/BS). In addition, we observed significant down-regulation of miR-29b-3p in patients undergoing anti-resorptive treatment compared to treatment-naïve patients. This suggests that high circulating levels of miR-29b-3p could be a surrogate biomarker to estimate bone formation and bone turnover rates in patients. This potential application is further substantiated by our recent observation that miR-29b-3p levels are positively correlated to P1NP and down-regulated in patients who had repeatedly suffered from bone fragility fractures^11^.

Another principle finding of this study is the positive correlation between circulating miR-550a-3p and MAR as well as BS/BV. Interestingly, the up-regulation of miR-550a-5p, the opposite strand in the mir-550a precursor miRNA, was previously reported as a putative novel biomarker of fracture-risk in postmenopausal and type-2 diabetic women with an inhibitory role during osteogenic differentiation^13^. In addition, a recent paper identified a relation between miR-550a-5p and vitamin D metabolism as well as bone mineral density^21^. To date, there is no information about the biological role of miR-550a-3p in the context of bone metabolism. However, several other miRNAs have been identified *in-vitro* to be functionally associated to bone metabolism and potentially to the development of bone diseases^19,22–25^. Differences in miRNA-levels between patients with and without prevalent low-traumatic fractures were previously reported^13,26^. Moreover, patients with recently sustained low-traumatic femoral fractures showed a significantly different miRNA expression when compared to healthy controls without fractures^12^.

Ultimately, a new biomarker candidate has evolved from this study - miR-324-3p. As with miR-29b-3p, so too does miR-324-3p show a significant positive association to both MAR and BFR/BS, but also MS/BS. In terms of microstructure, miR-324-3p showed a trend towards negative association to cortical porosity. ART significantly reduced serum levels of miR-324-3p compared to treatment-naïve patients. At this stage, little public information is available describing the biological functions of miR-324-3p, particularly in the context of bone metabolism. It is, however, of note that we have recently observed a down-regulation of miR-324 in serum of patients with recent osteoporotic fractures^12^, and that its serum levels are also positively correlated to bone mineral density at the spine^11^.

The assessment of differences in trabecular and cortical bone microstructure between male, premenopausal IOP as well as postmenopausal women with low-traumatic fractures was an additional objective of the present study. Deteriorations of trabecular and cortical bone microstructure are related to low-traumatic fractures in IOP and postmenopausal women, largely independent of aBMD^6–8,27,28^.

Cortical porosity is linked to age- and disease-related intra-cortical remodeling and was suggested to be a useful tool for fracture risk prediction^7,27^. The accuracy of detecting pores larger than 140 μm diameter was described to be excellent for non-invasive HR-pQCT^29^. However, about 60% of cortical pores are smaller than 100 μm and the bulk of pores cannot be detected by HR-pQCT resulting in a probable underestimation of porosity. In the present study using a more than ten-fold higher resolution than HR-pQCT even small pores were detected, resulting in a two-fold higher cortical porosity than previously described.

Although a higher cortical porosity was reported in male compared to female subjects and older compared to younger patients^30^, no significant differences were found between the groups in the present study. These results indicate no specific bone microstructure pattern in IOP. Moreover, the high variation of bone microstructure parameters reflects the heterogeneity of IOP^30^.

Both vertebral and peripheral fractures seem to be associated to trabecular and cortical bone^31,32^. Moreover, an association between impaired trabecular microstructure and cortical porosity was reported^7^. Trabecular bone microstructure was significantly impaired in patients with vertebral fractures and a higher cortical porosity by trend was found in patients with peripheral fractures (data not shown). As a proof of concept, highly significant correlations were found between the trabecular parameters by histomorphometry and µCT.

In addition to comparable bone microstructure, similar results regarding dynamic bone histomorphometry were seen among the three groups. This was somewhat unexpected, as a higher bone turnover was described in postmenopausal osteoporosis. In contrast, low bone formation parameters were reported in male and female IOP^2,3^. A likely explanation could be the differences in anti-resorptive therapy, which was more frequent in postmenopausal osteoporosis, probably resulting in low ES/BS and BFR/BS values compared to IOP.

Bearing in mind that neither bone mineral density, nor established bone turnover markers, nor FRAX, nor dynamic histomorphometry parameters explain the pathophysiology of IOP, diagnosis and fracture risk prediction in IOP is challenging^33^. Trabecular and cortical bone microstructure are impaired in IOP. That said, microstructure does not allow differentiation between premenopausal IOP, male IOP, and postmenopausal osteoporosis.

However, circulating miRNAs are promising new biomarkers for fracture risk prediction in IOP and might be used as tools for dissecting underlying pathomechanisms of IOP. A subset of miRNAs was recently reported to be an excellent discriminator of osteoporosis, independent of age and sex. Only a few, moderate correlations between these bone-related miRNAs and established bone turnover markers were found previously. Though miR-29b-3p was correlated to P1NP (procollagen type 1 N-terminal propeptide), no associations were found between CTX (β-crosslaps) and miRNAs^11^. These results suggest that circulating microRNAs can deliver additional information on bone metabolism which is not reflected by BTMs.

Here we have provided further data regarding the relationship between these miRNAs and bone microarchitecture and histomorphometry.

The limitation of this study was that correlations of microRNAs to µCT and histomorphometry parameters were based on an overall sample size of 36, which is not exceedingly high. Therefore, adjustment for multiple testing was not performed, which likely increases the number of false-positive associations. However, the purpose of this exploratory study was to gain additional information about circulating microRNAs with a known association to fracture risk. Another limitation is the lack of a control group. Performing bone biopsies in healthy controls is an invasive procedure and would not met the approval of the ethics committee. Despite this, differences in miRNA levels between male and premenopausal IOP, postmenopausal osteoporosis patients with low-traumatic fractures and healthy controls without fractures were reported in our previous study.

## Conclusion

Bone-related circulating miRNAs miR-29b-3p, miR-550a-3p and miR-324-3p are significantly associated to dynamic processes of bone, reflected by bone histomorphometry. This alludes to a putative causal relationship between these novel biomarker candidates and bone tissue. miRNAs miR-29b-3p, miR-550a-3p and miR-324-3p were recently found to discriminate patients with low-traumatic fractures and are therefore promising new biomarkers in the diagnosis of osteoporosis.

## Electronic supplementary material

Supplementary Information

Supplemental Data 1

Supplemental Table 1
