# Supplementary material for: Bone-related Circulating MicroRNAs miR-29b-3p, miR-550a-3p, and miR-324-3p and their Association to Bone Microstructure and Histomorphometry
Source: Sci Rep. 2018 Mar 20;8:4867. doi: 10.1038/s41598-018-22844-2 (PMC5861059; doi:10.1038/s41598-018-22844-2)
Supplement: Supplementary file 1 — Supplementary Information [file 41598_2018_22844_MOESM1_ESM.pdf]

Supplementary Information

Bone-related Circulating MicroRNAs miR-29b-3p, miR-550a-3p, and miR-324-3p and their Association to Bone Microstructure and Histomorphometry

Xaver Feichtinger[1,2,3], Christian Muschitz[2], Patrick Heimel[1,4,5], Andreas Baierl[6], Astrid Fahrleitner-Pammer[7], Heinz Redl[1,4], Heinrich Resch[2,8], Elisabeth Geiger[9], Susanna Skalicky[9], Rainer Dormann[2], Fabian Plachel[2], Peter Pietschmann[10], Johannes Grillari[9,11], Matthias Hackl[9], Roland Kocijan[1, 2]

1 Ludwig Boltzmann Institute for Experimental and Clinical Traumatology; Donaueschingenstraße 13, 1200 Vienna, Austria
2 St. Vincent Hospital – Medical Department II, The VINFORCE Study Group, Academic Teaching Hospital of the Medical University of Vienna, Austria
3 AUVA Trauma Center Meidling, Kundratstraße 37, 1120 Vienna, Austria
4 Austrian Cluster for Tissue Regeneration, Vienna, Austria Department of Traumatology, Medical University of Vienna, Austria
5 Karl Donath Laboratory for Hard Tissue and Biomaterial Research, Department of Oral Surgery, Medical University of Vienna, Austria
6 Department of Statistics and Operations Research, University of Vienna, Oskar Morgenstern-Platz 1, 1090 Vienna, Austria
7 Department of Internal Medicine, Division of Endocrinology and Diabetology, Medical University of Graz, Austria
8 Medical Faculty of Bone Diseases, Sigmund Freud University, Vienna, Sigmund Freud Platz 1
Austria
9 TAmiRNA GmbH, Muthgasse 18, 1190 Vienna, Austria
10 Department of Pathophysiology and Allergy Research, Center for Pathophysiology, Infectiology and Immunology, Medical University of Vienna, Austria
11 Department of Biotechnology, University of Natural Resources and Life Sciences Vienna, Austria

SUPPORTING FIGURES

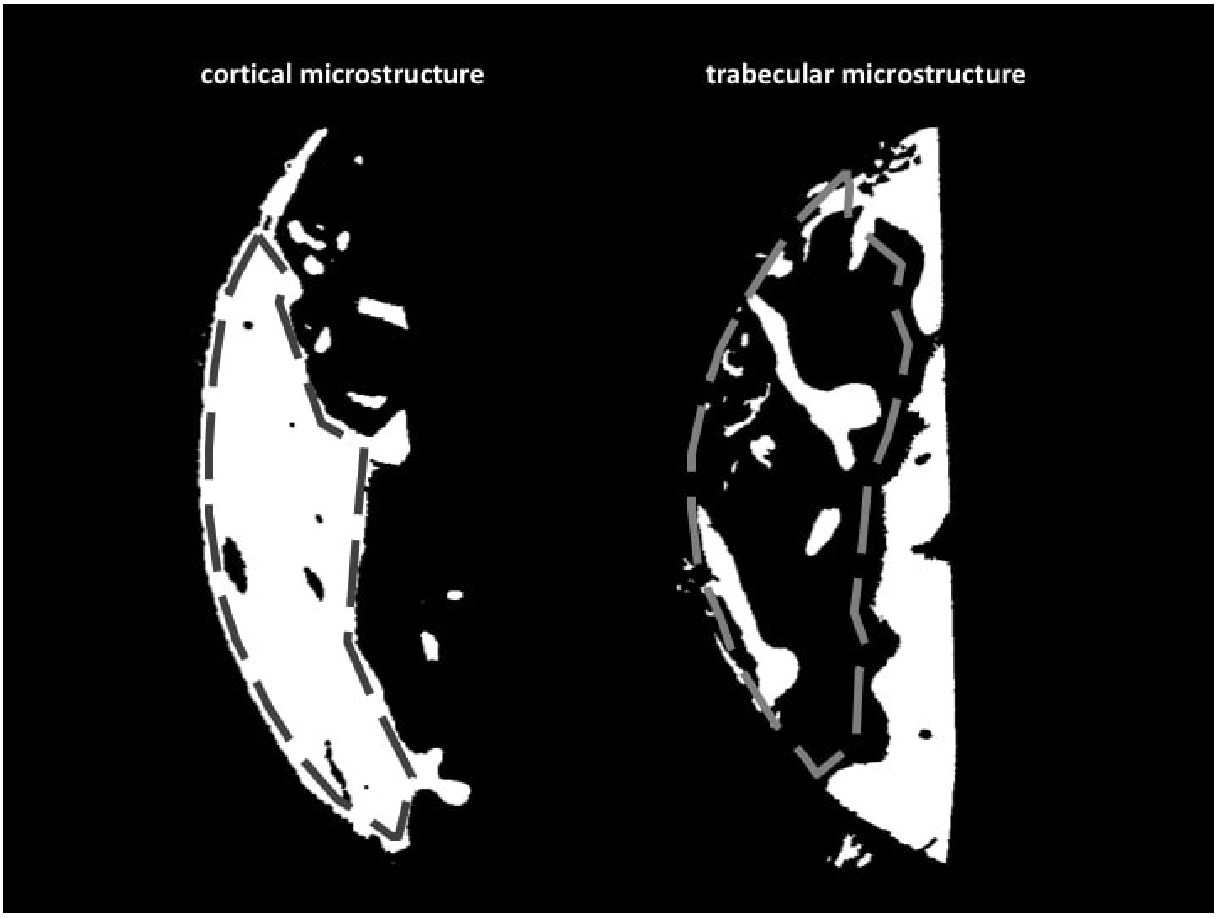

Supporting Figure 1. Segmentation and contouring of cortical and trabecular bone (dashed line).

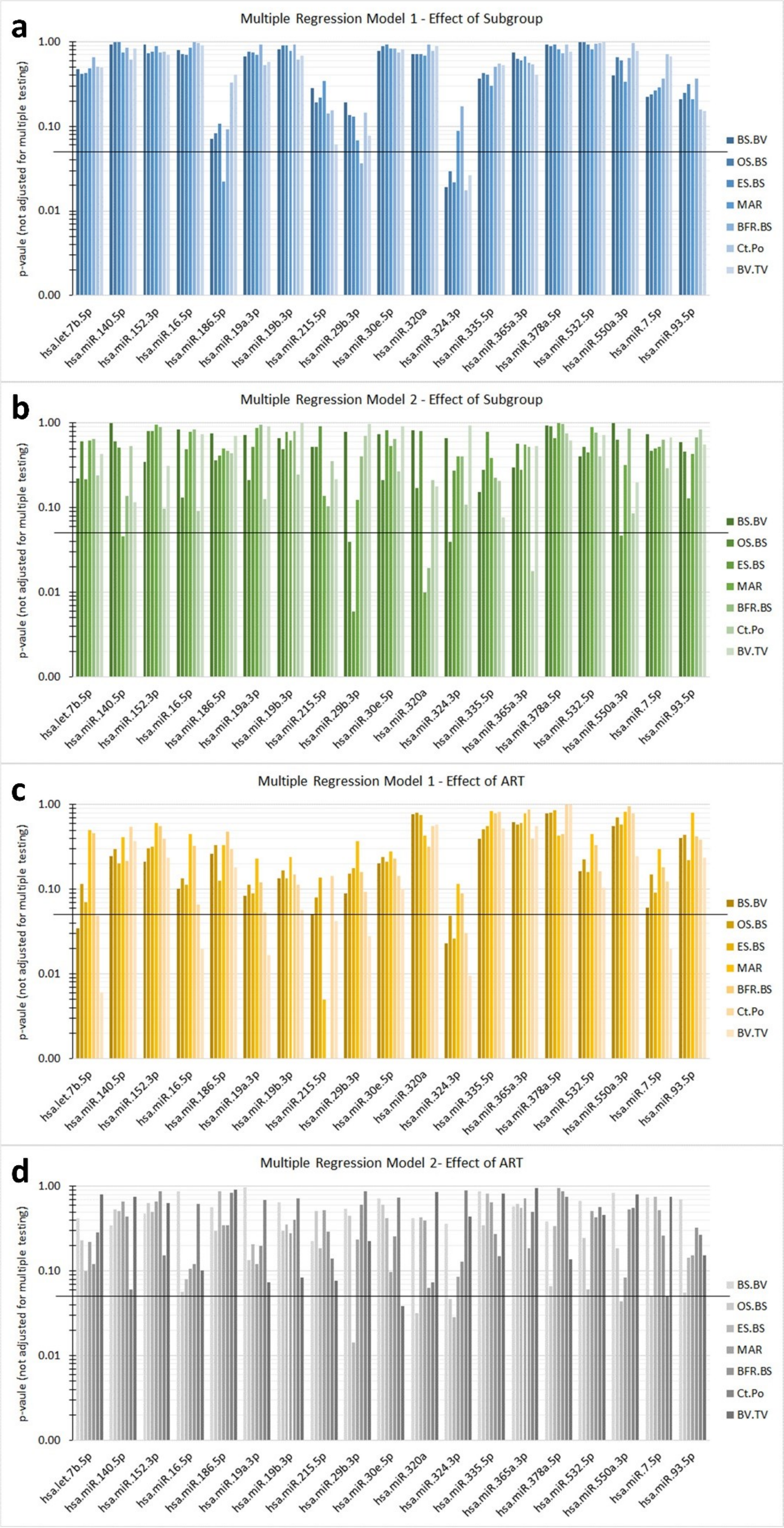
a
Multiple Regression Model 1 - Effect of Subgroup
b
Multiple Regression Model 2 - Effect of Subgroup
c
Multiple Regression Model 1 - Effect of ART
d
Multiple Regression Model 2- Effect of ART
p-vaule (not adjusted for multiple testing)
1.00
0.10
0.01
0.00
BS.BV
OS.BS
ES.BS
MAR
BFR.BS
Ct.Po
BV.TV
hsa.let.7b.5p
hsa.miR.140.5p
hsa.miR.152.3p
hsa.miR.16.5p
hsa.miR.186.5p
hsa.miR.19a.3p
hsa.miR.19b.3p
hsa.miR.215.5p
hsa.miR.29b.3p
hsa.miR.30e.5p
hsa.miR.320a
hsa.miR.324.3p
hsa.miR.335.5p
hsa.miR.365a.3p
hsa.miR.378a.5p
hsa.miR.532.5p
hsa.miR.550a.3p
hsa.miR.7.5p
hsa.miR.93.5p

Supporting Figure 2. Step-wise multiple regression analysis for 19 miRNAs against 7 bone parameters. P-values were obtained for two regression models assuming either offsets between the regression lines obtained for each subgroup (Model 1) or differences in the nature of regression (i.e. slope) between the subgroups (Model 2). P-values are shown for the effects of subgroup (premenopausal, postmenopausal, males) according to Model 1 (a) and Model 2 (b), and for the effects of anti-resorptive treatment (ART) according to multiple regression Model 1 (c) and Model 2 (d). Horizontal lines indicate a p-value threshold of 0.05. We conclude that the vast majority of associations are not affected by subgroup or ART.

Supporting Table 1. List of microRNAs analysed during this study.

#	mature ID	mature Seq	mature Acc
1	hsa-let-7b-5p	UGAGGUAGUAGGUUGUGUGGUU	MIMAT0000063
2	hsa-miR-140-5p	CAGUGGUUUUACCCUAUGGUAG	MIMAT0000431
3	hsa-miR-152-3p	UCAGUGCAUGACAGAACUUGG	MIMAT0000438
4	hsa-miR-16-5p	UAGCAGCACGUAAAUAUUGGCG	MIMAT0000069
5	hsa-miR-186-5p	CAAAGAAUUCUCCUUUUGGGCU	MIMAT0000456
6	hsa-miR-19a-3p	UGUGCAAAUCUAUGCAAAACUGA	MIMAT0000073
7	hsa-miR-19b-3p	UGUGCAAAUCCAUGCAAAACUGA	MIMAT0000074
8	hsa-miR-215-5p	AUGACCUAUGAAUUGACAGAC	MIMAT0000272
9	hsa-miR-29b-3p	UAGCACCAUUUGAAAUCAGUGUU	MIMAT0000100
10	hsa-miR-30e-5p	UGUAAACAUCCUUGACUGGAAG	MIMAT0000692
11	hsa-miR-320a	AAAAGCUGGGUUGAGAGGGCGA	MIMAT0000510
12	hsa-miR-324-3p	ACUGCCCCAGGUGCUGCUGG	MIMAT0000762
13	hsa-miR-335-5p	UCAAGAGCAAUAACGAAAAAUGU	MIMAT0000765
14	hsa-miR-365a-3p	UAAUGCCCCUAAAAAUCCUUAU	MIMAT0000710
15	hsa-miR-378a-5p	CUCCUGACUCCAGGUCCUGUGU	MIMAT0000731
16	hsa-miR-532-5p	CAUGCCUUGAGUGUAGGACCGU	MIMAT0002888
17	hsa-miR-550a-3p	UGUCUUACUCCCUCAGGCACAU	MIMAT0003257
18	hsa-miR-7-5p	UGGAAGACUAGUGAUUUUGUUGU	MIMAT0000252
19	hsa-miR-93-5p	CAAAGUGCUGUUCGUGCAGGUAG	MIMAT0000093
